# Treatment patterns, intensive treatment, and hospitalization-related burden of juvenile generalized myasthenia gravis in Japan: a retrospective cohort study using a hospital-based claims database

**DOI:** 10.3389/fneur.2026.1877931

**Published:** 2026-09-15

**Authors:** Keiko Ishigaki, Hiroshi Horio, Takahide Kitamura, Yosuke Saga

**Affiliations:** 1 Department of Pediatrics, Tokyo Women’s Medical University School of Medicine, Tokyo, Japan; 2 Medical Affairs Division, Johnson & Johnson, Tokyo, Japan

**Keywords:** claims database, corticosteroids, ICU hospitalization, intensive treatment, intravenous immunoglobulin, juvenile myasthenia gravis, tacrolimus, treatment pattern

## Abstract

**Objective:**

Juvenile generalized myasthenia gravis (MG) management aims to control symptoms while minimizing corticosteroid-related toxicity. However, large-scale evidence on real-world pediatric treatment patterns remains limited.

**Methods:**

We conducted a retrospective cohort study using a large Japanese hospital-based claims database (Medical Data Vision). We identified patients aged 0–17 years with juvenile generalized MG between April 2008 and November 2024. We summarized the use of cholinesterase inhibitors, oral corticosteroids, immunosuppressants, and intensive treatments (plasma exchange, intravenous immunoglobulin [IVIg], or high-dose steroid pulse therapy) during the one-year follow-up period after the first confirmed MG diagnosis recorded in the database. We also compared patient characteristics and treatment patterns by age group (children: 0–9 years vs. adolescents: 10–17 years).

**Results:**

We identified 261 patients with juvenile generalized MG, including 125 children and 136 adolescents cases. Cholinesterase inhibitors were the most frequently used medication (69.3% [181/261]). Oral corticosteroids were commonly used (65.9% [172/261]), whereas immunosuppressant use was limited; only 89/261 (34.1%) received tacrolimus. Among the 217 patients with complete one-year follow-up data, 146 received corticosteroid. The mean daily corticosteroid dose, calculated as the annual cumulative corticosteroid dose divided by 365 days, was 8.7 mg/day (SD 7.1) (children: 6.2 mg/day [SD 4.1]; adolescents: 11.4 mg/day [SD 8.6]). Among 261 generalized MG patients, 52 (19.9%) received intensive treatments, with a higher proportion in adolescents than children (25.7% [35/136] vs. 13.6% [17/125]). IVIg was the most common intensive treatment (13.4% [35/261]), followed by steroid pulse therapy (10.3% [27/261]) and plasma exchange (3.8% [10/261]). Adolescent generalized MG had higher intensive care unit hospitalization rates than child generalized MG (8.8% [12/136] vs. 0.8% [1/125]), and all thymectomies occurred in adolescent patients (8.1% [11/136] vs. 0% [0/125]).

**Conclusion:**

In Japanese real-world practice, about one in five children with generalized MG required intensive treatments, particularly among adolescents, while steroid-sparing immunosuppression was used in only one-third. These findings support close monitoring of adolescent generalized MG and highlight opportunities to optimize steroid-sparing management in juvenile generalized MG.

## 1 Introduction

Juvenile myasthenia gravis (MG) is an autoimmune disorder of the neuromuscular junction that presents distinct clinical and therapeutic challenges in children. Epidemiological data on its prevalence are limited; however, population-based studies suggest that early-onset MG accounts for a substantial proportion of all MG cases (1–4). The clinical presentation varies according to age at onset and geographic background (1, 4). Ocular MG is particularly common among younger children in Asian populations; in a Japanese nationwide survey, it accounted for 80.6% of cases with onset at 0–4 years and 61.5% of cases with onset at 5–9 years (2, 3). Early-onset and ocular disease have also been associated with a greater likelihood of remission (2, 5). In contrast, adolescent-onset juvenile MG patients tend to resemble adult-onset MG more closely (4, 5).

Treatment for juvenile MG is generally adapted from adult MG practice because pediatric trial data remain limited (2, 6). Although juvenile generalized MG is often considered to have a favorable prognosis relative to adult generalized MG, a subset of patients experience worsening of disease or refractory symptoms that require intensive treatment, including plasma exchange, intravenous immunoglobulin (IVIg), or high-dose intravenous steroid pulse therapy (6, 7). Cholinesterase inhibitors have long served as first-line symptomatic treatment for both adult and juvenile MG. While traditional therapies, such as oral corticosteroids and immunosuppressants, remain key components of current treatment (8, 9), recent targeted therapies have expanded the treatment landscape for adult patients. These novel agents have been suggested to decrease overall disease burden by providing rapid efficacy and reducing the burden of adverse effects associated with traditional treatment. For juvenile MG, these targeted therapies are highly anticipated future options, although the need for dedicated studies in this rare pediatric population remains strongly emphasized (10). Current treatment goals for adults recommend tapering corticosteroids to a maintenance dose of ≤5 mg/day prednisolone-equivalent, as doses above 7.5 mg/day are associated with long-term adverse effects (8, 11, 12). In children, however, prolonged corticosteroid therapy carries additional risks, such as growth failure, poor bone mineralization, Cushingoid features, and increased susceptibility to infection (6, 13). These side effects can disrupt school attendance and quality of life, and are a frequent reason families decline ongoing steroid therapy (13). Pediatric corticosteroid doses that appear modest in absolute terms (mg/day) may be relatively high when adjusted for body weight (mg/kg). Guidelines therefore recommend early initiation of steroid-sparing immunosuppressants in juvenile MG to reduce the cumulative corticosteroid burden (6, 9).

Nonetheless, how juvenile generalized MG is treated in daily clinical practice remains poorly characterized. Most available evidence comes from single-center case series or extrapolation from adult MG trials, and large-scale data on treatment patterns are limited (6, 8, 14). The field still lacks knowledge about whether corticosteroid dosing aligns with guideline targets, if steroid-sparing immunosuppressants are introduced early and used appropriately, and what proportion of patients require intensive treatment. This evidence gap is especially relevant in Japan, where child-onset juvenile MG is more common than in Western populations and treatment practices may differ (3).

Thus, using a large Japanese hospital-based claims database (Medical Data Vision), we conducted a retrospective cohort study to describe treatment patterns in patients with juvenile generalized MG in Japan. We examined corticosteroid dosing, immunosuppressant use, and the proportion of patients requiring intensive treatments, stratified by age group (children aged 0–9 years vs. adolescents aged 10–17 years).

## 2 Materials and methods

### 2.1 Data

We used the Medical Data Vision (MDV) database, a hospital-based claims database in Japan covering more than 400 acute care hospitals under the Diagnosis Procedure Combination (DPC) payment system. The database contains anonymized patient-level records, including demographics, International Classification of Diseases, 10th Revision (ICD-10) diagnoses, prescribed medications (Anatomical Therapeutic Chemical codes), procedures (Japan-specific K codes), laboratory tests, and hospitalization records. This study used claims data from April 1, 2008, through November 30, 2024 (*N* = 50,927,004).

The eligible population for this study comprised patients with generalized MG who were younger than 18 years. Patients with MG were identified using ICD-10 code G70.0, with at least two separate diagnosis records during the study period. We further required at least one MG-related treatment (oral corticosteroids, pyridostigmine, azathioprine, tacrolimus, cyclosporine) and at least one MG-related diagnostic test or procedure (anti-acetylcholine receptor or anti-muscle-specific kinase antibody testing, edrophonium [Tensilon] testing, thymectomy). The cohort was restricted to patients aged 0–17 years at the date of their first confirmed MG diagnosis, which was defined as the index date. We excluded patients with hepatitis B virus infection (ICD-10: B16, B18.1). MG subtype (ocular vs. generalized MG) was defined using the first MG-relevant ICD-10 code recorded at the index date, and only patients classified as generalized MG were included in the analysis (Figure 1). Patients were further classified into children (0–9 years) and adolescents (10–17 years) age groups based on age at the index date. This age-based stratification was used for descriptive comparison and was intended to distinguish younger children from adolescents, consistent with the World Health Organization definition of adolescence beginning at age 10, rather than to define biologically confirmed pubertal status.

**Figure 1:**
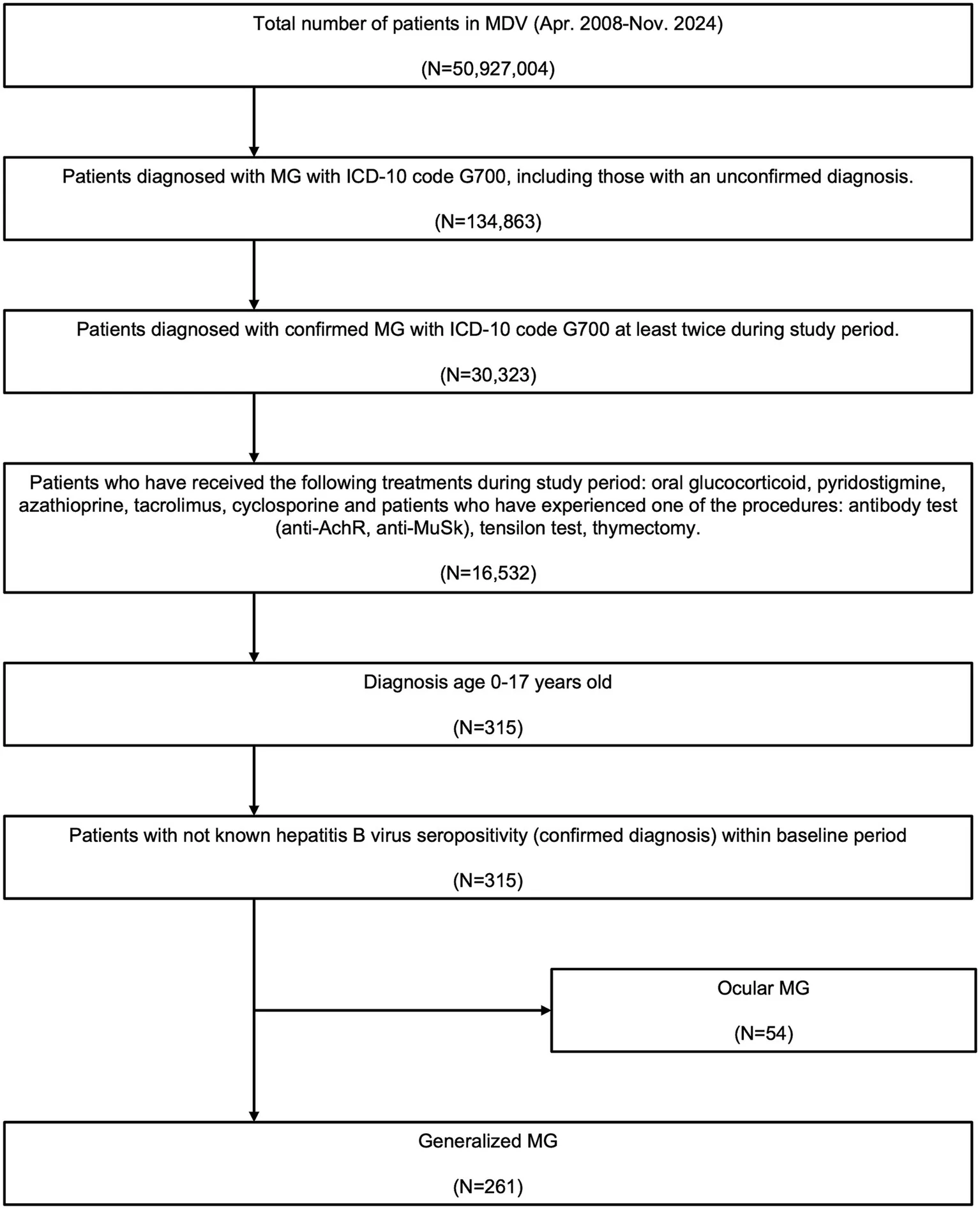
Patient selection flowchart. Selection of the juvenile generalized MG study cohort from the MDV hospital-based claims database. Anti-AChR, anti-acetylcholine receptor; anti-MuSK, anti-muscle-specific kinase; ICD-10, International Classification of Diseases, 10th Revision; MDV, Medical Data Vision; MG, myasthenia gravis.

Three study periods were defined relative to the index date: (1) a baseline period from the latest of the study start date, the patient’s first observation date, or 12 months before the index date, through the day before the index date; (2) a one-year follow-up period from the index date through 11 months afterward (or study end, death, or last available record, whichever came first); and (3) a whole follow-up period from the index date to study end, death, or last available record, with no fixed duration.

### 2.2 Measures

Patient characteristics assessed at the index date included age, age group (children aged 0–9 years vs. adolescents aged 10–17 years), and gender. Comorbidities included thymoma, hyperthyroidism, Hashimoto’s thyroiditis, rheumatoid arthritis, and systemic lupus erythematosus.

We recorded diagnostic tests performed during the baseline period and one-year follow-up period combined. These included autoantibody testing (anti-acetylcholine receptor and anti-muscle-specific kinase antibodies), chest computed tomography (CT), magnetic resonance imaging (MRI), repetitive nerve stimulation, edrophonium (Tensilon) testing, and single fiber electromyography.

Use of cholinesterase inhibitors, oral corticosteroids (prednisolone-equivalent mg/day), immunosuppressants (tacrolimus, cyclosporine, and others), and eculizumab were assessed at baseline and during follow-up. All pediatric corticosteroid doses are reported in absolute number of daily milligrams (mg/day). We estimated the daily dose (mg/day) by dividing the annual cumulative dosage amount by 365 days.

Intensive treatment was defined as receipt of plasma exchange (plasmapheresis), IVIg, or intravenous steroid pulse therapy (>1,000 mg/day) during follow-up. Clinical events recorded included intensive care unit (ICU) hospitalization, thymectomy, and respirator use.

### 2.3 Statistical analysis

All analyses were descriptive. Continuous variables were reported as means with standard deviations (SD) and medians; categorical variables were reported as counts and percentages.

Patient demographics and clinical characteristics were summarized for the overall cohort (all eligible juvenile generalized MG patients, irrespective of follow-up duration) and stratified by age group (children aged 0–9 years vs. adolescents aged 10–17 years). These summaries covered the baseline period, the one-year follow-up period, and both periods combined.

Treatment exposure was summarized for the overall, children, and adolescent groups. Mean differences and 95% confidence intervals (CIs) were calculated for selected continuous treatment variables between the children and adolescent groups. For the subcohort of patients with ≥1 year of follow-up, the treatment sequence in the whole follow-up period was additionally assessed. Treatment sequence analyses described the first, second, and third treatments by drug class (cholinesterase inhibitors, oral corticosteroids, tacrolimus, cyclosporine, eculizumab, and other immunosuppressants) over the whole follow-up period for this subcohort.

Missing values were not imputed. For continuous variables, analyses were restricted to patients with available data (complete case analysis). Binary variables (e.g., treatment use, comorbidity presence) were determined by the presence of a corresponding claim record; absence of a record was treated as non-occurrence rather than missing data. All analyses were performed using SAS version 9.4 or later (SAS Institute, Cary, NC).

This study used anonymized claims data with no identifiable patient information. Patient identifiers had been removed from the dataset before release to investigators. Under the Japanese Ethical Guidelines for Epidemiological Research, studies using anonymized secondary data are exempt from institutional ethics review and the requirement for individual informed consent.

## 3 Results

Table 1 shows demographic and clinical characteristics of patients with generalized MG overall and by age group. Mean age at diagnosis was 9.4 years (SD 5.6; range 0–17), and the age groups were distributed almost equally between children aged 0–9 years (125, 47.9%) and adolescents aged 10–17 years (136, 52.1%). Females comprised 63.2% (165/261) of the overall cohort and 64.7% (88/136) of the adolescents group. The mean follow-up duration was 332.2 days (SD 84.7).

**Table 1:** Demographic and clinical characteristics overall and by age group.

Patient demographic and clinical characteristics	Overall	By age group	
		Children (0–9 years)	Adolescents (10–17 years)
	(*N* = 261)	(*N* = 125)	(*N* = 136)
Age^*^ (years)			
Mean (SD)	9.4 (5.6)	4.1 (2.8)	14.3 (2.1)
Median (range)	10 (0–17)	4 (0–9)	15 (10–17)
Gender^*^			
Male	96 (36.8)	48 (38.4)	48 (35.3)
Female	165 (63.2)	77 (61.6)	88 (64.7)
Follow-up period (days)			
Mean (SD)	332.2 (84.7)	334.9 (82.0)	329.7 (87.4)
Median (range)	365 (37–366)	365 (45–366)	365 (37–366)
Intensive treatment	52 (19.9)	17 (13.6)	35 (25.7)
Plasmapheresis	10 (3.8)	1 (0.8)	9 (6.6)
IVIg	35 (13.4)	13 (10.4)	22 (16.2)
Steroid pulse therapy	27 (10.3)	4 (3.2)	23 (16.9)
Clinical events			
ICU hospitalization	13 (5.0)	1 (0.8)	12 (8.8)
Thymectomy	11 (4.2)	0 (0.0)	11 (8.1)
Use of respirator	1 (0.4)	1 (0.8)	0 (0.0)
Comorbidities			
Thymoma	15 (5.7)	2 (1.6)	13 (9.6)
Hyperthyroidism	8 (3.1)	1 (0.8)	7 (5.1)
Hashimoto’s thyroiditis	2 (0.8)	0 (0.0)	2 (1.5)
Rheumatoid arthritis	2 (0.8)	0 (0.0)	2 (1.5)
Systemic lupus erythematosus	2 (0.8)	0 (0.0)	2 (1.5)
Diagnostic tests			
Autoantibody test	254 (97.3)	123 (98.4)	131 (96.3)
Chest CT	105 (40.2)	40 (32.0)	65 (47.8)
MRI	107 (41.0)	49 (39.2)	58 (42.6)
Repetitive nerve stimulation	112 (42.9)	52 (41.6)	60 (44.1)
Edrophonium testing (Tensilon test)	121 (46.4)	67 (53.6)	54 (39.7)
Single fiber electromyography	0 (0.0)	0 (0.0)	0 (0.0)

Demographic and clinical characteristics of patients with juvenile generalized MG, overall and stratified by age group. Age and gender were assessed at the index date (the date of first confirmed MG diagnosis). Diagnostic tests were assessed over the combined baseline and follow-up periods, whereas the other variables (intensive treatment, clinical events, and comorbidities) were assessed during the one-year follow-up period only. Intensive treatment was defined as plasma exchange, IVIg, or high-dose intravenous steroid pulse therapy. Data are presented as *n* (%) unless otherwise indicated.

CT, computed tomography; ECG, electrocardiogram; ICU, intensive care unit; IVIg, intravenous immunoglobulin; MG, myasthenia gravis; MRI, magnetic resonance imaging; SD, standard deviation.

Diagnostic testing was performed in most patients during the baseline period and/or the one-year follow-up period. Autoantibody testing was conducted in 97.3% of the cohort. Repetitive nerve stimulation was used in 42.9%, while edrophonium (Tensilon) testing was performed in 46.4%. No patient underwent a single fiber electromyography.

Comorbidities occurred more frequently in adolescent patients. Thymoma was present in 15/261 (5.7%) overall, including 9.6% in adolescent patients and 1.6% in child patients. Eight patients (3.1%) had hyperthyroidism, corresponding to 5.1% of adolescent versus 0.8% of child patients. Other autoimmune conditions (Hashimoto’s thyroiditis, rheumatoid arthritis, systemic lupus erythematosus) each occurred in 0.8% of the cohort. Nearly all autoimmune comorbidities occurred among adolescent patients.

Table 2 shows treatment exposure profile of the overall cohort. Details of treatment trajectories over the follow-up period, including treatment order, are detailed in Supplementary Table 1 and visualized with Sankey diagram in Figure 2. Cholinesterase inhibitors were used in 181/261 (69.3%) of the cohort. Oral corticosteroids were prescribed to 172/261 (65.9%) patients; usage rates were similar across age groups (children 70.4% vs. adolescents 61.8%). Among patients who received oral corticosteroids (*n* = 172), the mean initial prednisolone-equivalent dose was 15.5 mg/day, and adolescent patients initiated corticosteroids at a higher dose than child patients (19.9 mg/day versus 11.4 mg/day).

**Table 2:** Treatment exposure overall and by age group.

Treatment exposure and dosing		Overall	By age group		Mean difference (children - adolescents)	95% CI
			Children (0–9 years)	Adolescents (10–17 years)		
		(*N* = 261)	(*N* = 125)	(*N* = 136)		
Cholinesterase inhibitors		181 (69.3)	83 (66.4)	98 (72.1)	-	-
Oral corticosteroid^*^		172 (65.9)	88 (70.4)	84 (61.8)	-	-
Initial dose of oral corticosteroid^*^ (mg/day)	*n*	172	88	84		
	Mean (SD)	15.5 (15.8)	11.4 (7.2)	19.9 (20.5)	−8.5	−13.2, −3.8
	Min–max	0.5–125.0	0.5–35.0	1.0–125.0		
Maximum dose of oral corticosteroid^*^ (mg/day)	*n*	172	88	84		
	Mean (SD)	29.8 (31.7)	20.4 (16.8)	39.6 (39.8)	−19.2	−28.5, −9.8
	Min–max	0.5–230.0	0.5–100.0	1.0–230.0		
Duration of administration of oral corticosteroid (days)	*n*	172	88	84		
	Mean (SD)	244.0 (110.1)	241.6 (110.1)	246.5 (110.8)	−4.9	−38.1, 28.4
Mean daily prescription dose for 1 year (mg/day) ^*†^ (among oral corticosteroid users with complete one-year follow-up)	*n*	146	77	69		
	Mean (SD)	8.7 (7.1)	6.2 (4.1)	11.4 (8.6)	−5.2	−7.4, −2.9
	Median	7.1	6.1	9.4		
	Min–max	0.02–40.8	0.02–16.3	0.19–40.8		
Immunosuppressants		91 (34.9)	38 (30.4)	53 (39.0)	-	-
Tacrolimus		89 (34.1)	38 (30.4)	51 (37.5)	-	-
Cyclosporine		2 (0.8)	0 (0.0)	2 (1.5)	-	-
Other immunosuppressants		4 (1.5)	0 (0.0)	4 (2.9)	-	-
Azathioprine		0 (0.0)	0 (0.0)	0 (0.0)	-	-
Initial dose of tacrolimus (mg/day)	*n*	89	38	51		
	Mean (SD)	1.9 (0.9)	1.4 (0.8)	2.2 (0.8)	−0.9	−1.2, −0.5
	Min–max	0.2–3.0	0.3–3.0	0.2–3.0		
Maximum dose of tacrolimus (mg/day)	*n*	89	38	51		
	Mean (SD)	2.6 (1.2)	1.9 (0.8)	3.2 (1.2)	−1.2	−1.7, −0.8
	Min–max	0.2–6.0	0.4–4.0	0.2–6.0		

Treatment exposure during the one-year follow-up period among patients with juvenile generalized MG, overall and stratified by age group. Medication use is reported for any exposure during follow-up, and dose-related variables are summarized among treated patients only. Oral corticosteroid doses are expressed as prednisolone-equivalent doses. These values refer to oral corticosteroid prescriptions and do not include intravenous steroid pulse therapy. Among 217 patients with complete one-year follow-up, 146 oral corticosteroid users were included in the dose analyses. Mean daily corticosteroid dose was calculated as the cumulative one-year corticosteroid dose divided by 365 days among patients with complete one-year follow-up. Mean differences are shown as children minus adolescents values, with 95% CIs.

^*^Prednisolone-equivalent amount.

^†^365 days from the date of the first confirmed diagnosis of MG (index date).

CI, confidence interval; MG, myasthenia gravis; SD, standard deviation.

**Figure 2:**
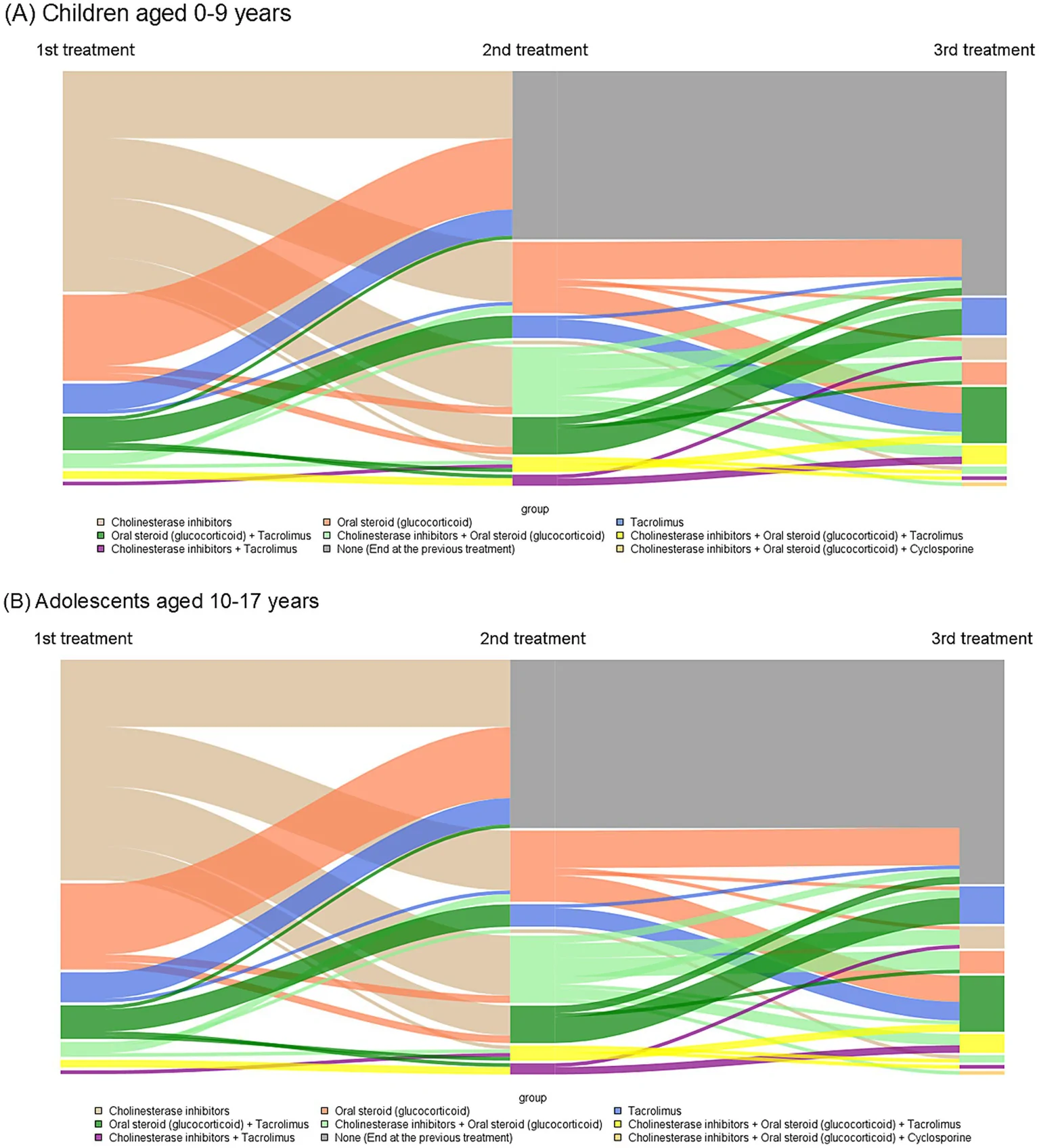
Sankey diagrams showing treatment trajectory over the entire follow-up period among patients with ≥1 year of follow-up for children aged 0–9 years **(A)** and adolescents aged 10–17 years **(B)**. The three horizontal columns (1st, 2nd, and 3rd treatments) illustrate the chronological sequence of treatment modifications rather than specific intervals. The gray area indicates the discontinuation of treatments.

Among oral corticosteroid users with complete one-year follow-up (*n* = 146), the mean daily corticosteroid dose, calculated by dividing the cumulative annual corticosteroid dose by 365 days, was 8.7 mg/day (SD 7.1). Adolescent patients maintained higher doses with the mean difference of −5.2 (95% CI: −7.4 to −2.9) mg/day (11.4 mg/day [SD 8.6] versus 6.2 mg/day [SD 4.1]).

Immunosuppressants were prescribed to 91/261 (34.9%) patients. Tacrolimus was the primary immunosuppressant, prescribed to 89/261 (34.1%) patients (children 30.4% vs. adolescents 37.5%). Among patients prescribed “other” immunosuppressants, no one received azathioprine in either age group. Mean initial dose of tacrolimus was 1.9 mg/day (SD 0.9; children 1.4 mg/day [SD 0.8] vs. adolescents 2.2 mg/day [SD 0.8]), mean maximum dose was 2.6 mg/day (SD 1.2). Cyclosporine was rarely used (2/261, 0.8%), and eculizumab was administered to one patient (0.4%).

Figure 3 shows the proportion of patients receiving intensive treatment (plasma exchange, IVIg, or steroid pulse therapy) during the one-year follow-up period in the overall cohort and in the child and adolescent subgroups. About one in five patients (52/261, 19.9%) received intensive treatment. A higher proportion of adolescent patients received intensive treatment than child patients (35/136, 25.7% vs. 17/125, 13.6%). Among patients undergoing both thymectomy and intensive treatment (*N* = 11), four received intensive therapy before surgery, one on the same day, and three within 30 days following thymectomy. Patients who fall into multiple categories are counted more than once. Of the 31 patients who received both intensive treatment and immunosuppressants, 11 initiated immunosuppressants before intensive therapy, three on the same date, and 17 after.

**Figure 3:**
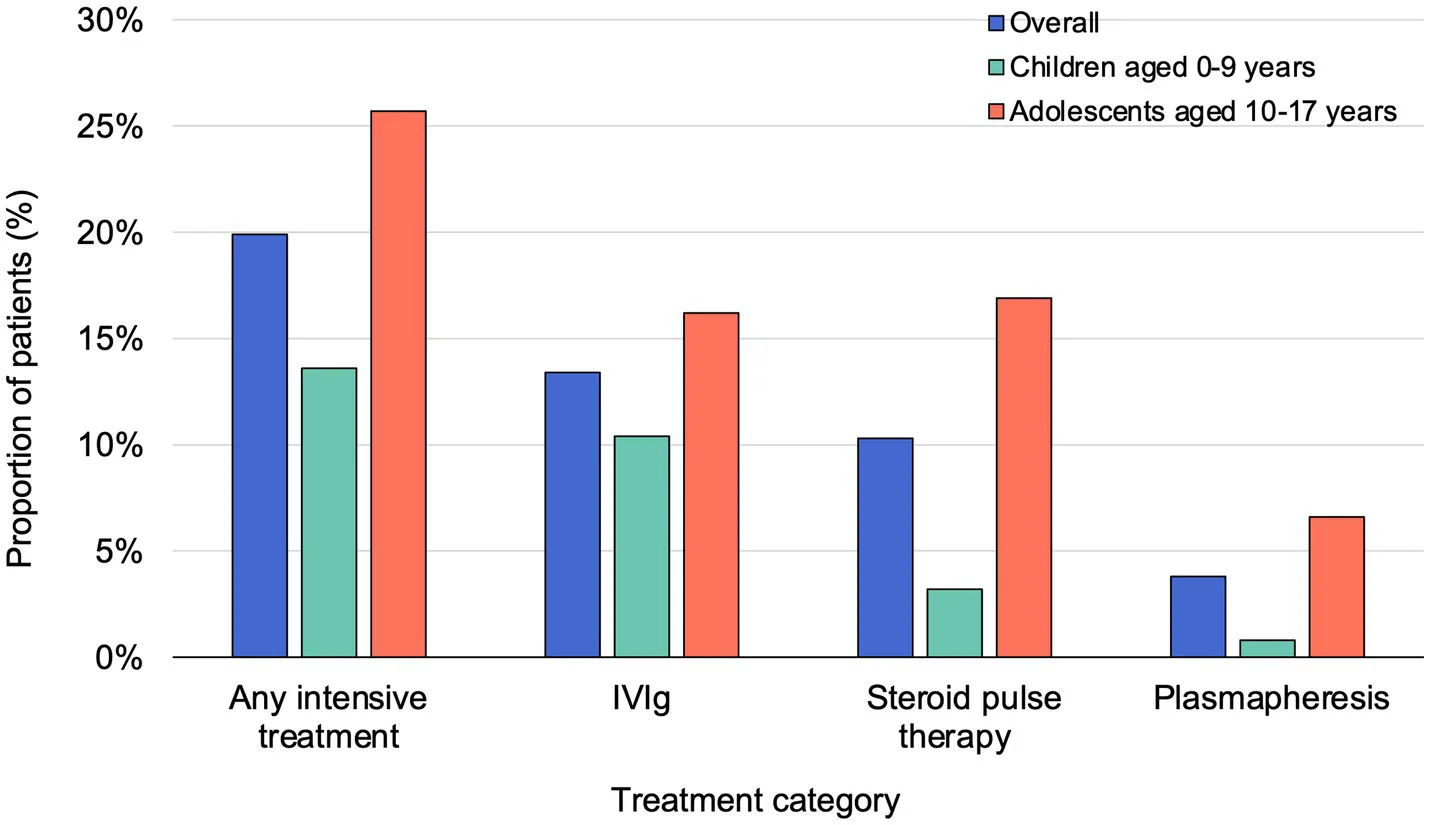
Proportion of patients receiving intensive treatment. Proportion of patients with juvenile generalized MG who received intensive treatment during the one-year follow-up period in the overall cohort and in the child (0–9 years) and adolescent (10–17 years) subgroups. Intensive treatment was defined as receipt of plasma exchange (plasmapheresis), IVIg, or high-dose intravenous steroid pulse therapy. Bars show the proportion of the generalized MG cohort receiving any intensive treatment and each specific intensive-treatment modality. Categories are not mutually exclusive. IVIg, intravenous immunoglobulin; MG, myasthenia gravis.

IVIg was the most frequently used intensive treatment in generalized MG, administered to 35/261 (13.4%) patients (children 10.4% vs. adolescents 16.2%). Steroid pulse therapy was the second most frequent, administered to 27/261 (10.3%; children 3.2% vs. adolescents 16.9%), and plasma exchange was used in 10/261 (3.8%; children 0.8% vs. adolescents 6.6%).

Other markers of disease severity were likewise more common among adolescent patients. ICU hospitalization occurred in 13/261 (5.0%) patients overall, with marked divergence between adolescent (12/136, 8.8%) and child (1/125, 0.8%) groups. Thymectomy was performed in 11/261 (4.2%) patients, exclusively in the adolescent group. Respirator use was recorded in one out of 261 generalized MG patients (0.4%), who was a child patient.

Table 3 shows treatment exposure in the intensive treatment subcohort. There were 53 generalized MG patients who received intensive treatment during the baseline period and/or the one-year follow-up period. Of these, 52 patients received intensive treatment during the one-year follow-up period (as reported in Table 1). Among intensive treatment patients, initial corticosteroid dose was comparable to the overall cohort (mean 14.8 mg/day [SD 11.8]), whereas the mean maximum dose was 41.7 mg/day (SD 42.6), higher than in the overall cohort (29.8 mg/day [SD 31.7]). The higher maximum dose was particularly pronounced in adolescent patients (50.3 mg/day [SD 49.7] vs. 25.6 mg/day [SD 14.9] in child patients). The use of immunosuppressants was limited even in this subcohort, with 31 of 53 (58.5%) patients having received an immunosuppressant. Supplementary Table 1 shows treatment patterns in patients with generalized MG. Cholinesterase inhibitors remained the most common first-line treatment (53.7%), while the most frequent second-line regimen combined a cholinesterase inhibitor with oral corticosteroids.

**Table 3:** Treatment exposure in the intensive treatment subcohort.

Intensive treatment characteristics		Overall	By age group		Mean difference (children - adolescents)	95% CI
		Overall	Children (0–9 years)	Adolescents (10–17 years)		
		(*N* = 53)	(*N* = 18)	(*N* = 35)		
Cholinesterase inhibitors		40 (75.5)	15 (83.3)	25 (71.4)	-	-
Oral corticosteroid*		49 (92.5)	17 (94.4)	32 (91.4)	-	-
Initial dose of oral corticosteroid (mg/day)*	*n*	49	17	32	−4.3	−10.2, 1.7
	Mean (SD)	14.8 (11.8)	12.0 (7.4)	16.3 (13.4)		
	Median	10.0	10.0	10.0		
	Min–max	2.5–60.0	4.0–27.0	2.5–60.0		
Maximum dose of oral corticosteroid (mg/day)*	*n*	49	17	32	−24.7	−43.9, −5.5
	Mean (SD)	41.7 (42.6)	25.6 (14.9)	50.3 (49.7)		
	Median	30.0	22.5	35.0		
	Min–max	5.0–230.0	9.0–54.0	5.0–230.0		
Immunosuppressants		31 (58.5)	9 (50.0)	22 (62.9)		-
Tacrolimus		31 (58.5)	9 (50.0)	22 (62.9)	-	-
Cyclosporine		0 (0.0)	0 (0.0)	0 (0.0)	-	-
Other immunosuppressants		4 (7.5)	0 (0.0)	4 (11.4)	-	-
Initial dose of tacrolimus (mg/day)	*n*	31	9	22	−1.4	−2.1, −0.7
	Mean (SD)	2.0 (1.0)	1.0 (0.9)	2.4 (0.6)		
	Median	2.0	0.5	2.8		
	Min–max	0.3–3.0	0.3–3.0	1.0–3.0		

Treatment exposure in the intensive-treatment subcohort of patients with juvenile generalized MG, overall and stratified by age group. The intensive-treatment subcohort comprised patients who received plasma exchange, IVIg, or high-dose intravenous steroid pulse therapy during the baseline period and/or the one-year follow-up period. Medication use is reported for any exposure within this subcohort, and dose-related variables are summarized among treated patients only. Oral corticosteroid doses are expressed as prednisolone-equivalent doses. Mean differences are shown as children minus adolescents values, with 95% CIs. The subcohort size of 53 includes one patient who received intensive treatment during baseline and therefore is not included among the 52 patients counted as receiving intensive treatment during the one-year follow-up period.

^*^Prednisolone-equivalent amount.

CI, confidence interval; IVIg, intravenous immunoglobulin; MG, myasthenia gravis; SD, standard deviation.

Table 4 shows hospitalization categories within the 1-year follow-up period for patients with generalized MG, stratified by age group. A total of 135 patients experienced at least one hospitalization, including 73 children and 62 adolescents. Among these hospitalized patients, 50 (37.0%) had at least one hospitalization associated with intensive treatment. Details of hospitalization summaries for each treatment type are presented in Supplementary Table 2.

**Table 4:** Hospitalization summary and classifications within one-year follow up period for generalized MG patients.

Hospitalization outcomes	All	Children (0–9 years)	Adolescent (10–17 years)
Number of target cases	261	125	136
Number of patients with hospitalization	135 (51.7)	73 (58.4)	62 (45.6)
Hospitalization category (number of patients)			
Any intensive treatment-associated hospitalization	50 (19.2)	16 (12.8)	34 (25.0)
IVIg-associated hospitalization	34 (13.0)	12 (9.6)	22 (16.2)
Plasma exchange-associated hospitalization	10 (3.8)	1 (0.8)	9 (6.6)
Steroid pulse therapy-associated hospitalization	25 (9.6)	4 (3.2)	21 (15.4)
ICU/respirator-associated hospitalization	13 (5.0)	1 (0.8)	12 (8.8)
Other hospitalization without intensive treatment, ICU, or respirator	101 (38.7)	67 (53.6)	34 (25.0)

Hospitalization categories are not mutually exclusive. Percentages are calculated using the number of target cases as the denominator.

ICU, intensive care unit; IVIg, intravenous immunoglobulin.

## 4 Discussion

In this retrospective cohort study of 261 children with generalized MG identified from a large Japanese hospital-based claims database, we found three key patterns in treatment during the first year after diagnosis. First, a substantial proportion of patients with generalized MG required intensive treatment. About one in five received IVIg, steroid pulse therapy, or plasmapheresis. Second, among adolescent patients, for whom treatment strategies similar to adults are recommended, corticosteroid doses were high (e.g., mean initial dose 19.9 mg/day), although the commonly cited ≤5 mg/day prednisolone-equivalent target is an adult treatment goal rather than a pediatric-specific threshold. Third, immunosuppressant use was limited, with tacrolimus prescribed in only about one-third of patients, suggesting that optimizing immunosuppressant use may improve disease control in juvenile generalized MG patients.

The proportion of generalized MG patients receiving intensive treatment (19.9%) is higher than expected, given that previous studies have shown that juvenile MG is relatively mild compared to adult MG (6, 7). Part of this elevated rate may be explained by temporary intensive therapies administered around the time of thymectomy. Although three of the 11 patients who underwent both procedures received intensive treatment within 30 days following thymectomy, the nature of claims data limits our ability to establish a direct causal link between the procedure and subsequent intensive therapy. When stratified by age groups, the difference between child and adolescent patients suggests distinct clinical courses. Child patients had a lower rate of intensive treatment (13.6%), consistent with reports of higher spontaneous remission rates (5, 15). Adolescent patients received intensive treatment at a higher rate (25.7%), more closely resembling treatment patterns in adult MG. This higher rate may partly reflect the greater physical tolerability of intensive treatments in older children, as well as potentially higher disease severity, given that adolescent-onset MG is known to present a clinical course closer to adult-onset disease. Our results may also reflect the fact that nearly half of our study population were adolescent patients, whose disease trajectory may be more similar to that of adult MG. Although the current study did not formally define severe exacerbations, which are commonly operationalized in adult MG studies as MG exacerbations, myasthenic crises, intravenous immunoglobulin administration, and plasmapheresis (16), we found that 5.0% of all patients with generalized MG and 8.8% of the adolescent subgroup required ICU admission. In addition, all thymectomies were performed in adolescent patients, corresponding to 8.1% of the adolescent generalized MG group. These findings suggest the presence of a subgroup with potentially severe disease and possible myasthenic crises. Notably, these observations align with US registry data (17), which also highlighted that adolescent groups more frequently required ICU admission, thymectomy, and rapid treatment intensification to later-line therapies compared with the child group. In adult generalized MG, first-year myasthenic crisis has been reported to be more frequent among patients who did not achieve minimal manifestation status at 24 months (18). Similarly, recent studies with the US registry data (17) and the UK cohort data (19) underscored that in juvenile patients, disease exacerbations and related hospitalizations are concentrated within the first year of diagnosis, suggesting a need of careful disease management in this time window. To further clarify treatment patterns, we summarized overall hospitalization profiles. Although claims data cannot easily differentiate between admissions for acute symptom worsening and those scheduled for routine treatment administration (e.g., elective IVIg), patients requiring highly intensive care, such as ICU admission, represent a critical subgroup. Identifying these severe events is crucial, as acute crises and exacerbations dramatically drive clinical and economic burdens; indeed, pediatric MG patients experiencing such acute events have been reported to incur approximately threefold higher healthcare costs compared to those without (20). Our finding that a notable proportion of adolescent patients experienced possible crises warrants further investigation. Taken together, findings support the need for close monitoring and preparedness for intensive treatment in juvenile generalized MG, particularly in adolescent patients.

Oral corticosteroids were commonly used in generalized MG, with 172 of 261 (65.9%) patients receiving them during the first year. In children, doses that appear modest in mg/day may be high when adjusted for body weight, and long-term corticosteroid exposure is associated with growth failure, bone loss, and effects on school life and quality of life (5, 13). Our findings on these oral treatment patterns and clinical trajectories in Japanese patients with juvenile MG are consistent with recent global reports. Aligning with previous international studies (17, 19, 21), we found that more than half of the patients received cholinesterase inhibitors as their first-line treatment.

Immunosuppressant use during the one-year follow-up period after the first definitive diagnosis was limited, with only 34.1% of generalized MG patients receiving tacrolimus, which may be lower than optimal if steroid-sparing is a priority. The current Japanese guideline for MG recommends proactive use of steroid-sparing immunosuppressants to avoid corticosteroid side effects in patients who become steroid-dependent (6, 9, 22). A comprehensive meta-analysis from other countries also supported that tacrolimus may improve symptoms of juvenile MG refractory to corticosteroids (21). Despite high overall steroid dependence, tacrolimus use among patients with generalized MG was only 34.1% in the overall cohort and 58.5% even in the intensive-treatment subcohort in our data.

Although the current Japanese guidelines do not provide clear indications for the use of second-line immunosuppressants in children, they suggest the use of tacrolimus to reduce steroid dependence in pediatric patients with steroid-resistant or steroid-dependent generalized MG (22). Expert opinion also frequently supports the use of azathioprine for juvenile generalized MG (6). Under the Japanese regulatory framework, however, the approved dosage regimen for tacrolimus in MG is specified only for adults, whereas azathioprine is not formally approved for MG. This regulatory context may partly explain the limited use of these agents in our cohort. Although pediatric off-label prescriptions are not subject to reimbursement deductions in Japan (23), the absence of a formal indication for azathioprine may still influence its use. Further enforcement of recommendations may aid the optimal use of nonsteroidal immunosuppressants including but not limited to azathioprine. Earlier and more consistent immunosuppressant introduction may help reduce long-term steroid exposure, which is particularly important in children.

The psychosocial influence of intensive therapies beyond clinical remission also warrants attention. Previous studies show that the side effects of high-dose corticosteroids, such as weight gain and mood disturbance, particularly affect the psychological well-being of adolescent MG patients (24). Beyond corticosteroids, intensive treatments such as IVIg, plasma exchange, and steroid pulse therapy carry their own physical burden and often require hospitalization, disrupting school life and daily activities and placing additional demands on caregivers. Intensive medical treatment in general has a profound mental burden on infants and children, with a meta-analysis indicating the presence of post-traumatic stress disorder among 10–30% of pediatric ICU survivors (25). Among adult MG patients, the prevalences of depression and anxiety were both above 30%, being higher than in the general population. Although no studies to date have assessed the mental health burden in juvenile MG, the mental health of this population—particularly given factors such as inability to attend school and the side effects of intensive therapy—warrants attention (26).

This study has limitations inherent to its claims-based design. First, identification of juvenile generalized MG relied on ICD-10 coding and claims for MG-related tests and treatments; miscoding may have resulted in misclassification of ocular MG as generalized MG. In addition, disease onset prior to the first confirmed diagnosis in the database may lack precision if the actual first diagnosis was made before the date captured in the database. Treatments and events occurring outside participating hospitals may also have been missed. Second, key clinical variables (e.g., Myasthenia Gravis Foundation of America class, Quantitative Myasthenia Gravis score, Myasthenia Gravis Activities of Daily Living score, antibody titers, body weight for mg/kg dosing, and detailed adverse events such as growth and bone outcomes) were unavailable. Severity and exacerbations were therefore inferred using proxies, such as ICU hospitalization and receipt of IVIg, plasmapheresis, or steroid pulse therapy. Third, the use of MDV data and the hospital-based sampling frame limits generalizability. The data likely overrepresent patients with more severe juvenile generalized MG who required hospital-based assessment or treatment, so the results may not be generalizable to patients managed in clinics or other hospitals. Fourth, the database did not record the indication for admission and therefore did not distinguish hospitalization for worsening MG symptoms from hospitalization for treatment administration such as IVIg. We analyzed ICU hospitalization, which is unlikely to represent admission solely for treatment administration, but the clinical reason for ICU admission could not be confirmed from claims. Sixth, although we specifically identified patients using the specific ICD-10 code for myasthenia gravis (G70.0) and required the initiation of standard MG therapies, the cohort diagnosed within the first two years of life might still include a very small number of patients with congenital myasthenic syndromes. Clinically, these genetic syndromes are managed with distinct medications (e.g., ephedrine or quinidine) that were not captured by our inclusion criteria, meaning any potential contamination is expected to be minimal. Finally, the maximum recorded daily dose of oral corticosteroids in our data was extremely high, which likely reflects potential information bias. This could have occurred if a multi-day supply of oral corticosteroids was recorded as a single daily dose at the time of prescription.

## 5 Conclusion

In real-world practice in Japan, approximately one in five patients with juvenile generalized MG required intensive treatment (IVIg, plasmapheresis, or steroid pulse therapy), and generalized MG in adolescents had a higher proportion of patients receiving intensive treatment than children, consistent with the possibility of higher disease severity in adolescent patients. ICU admission and thymectomy were also concentrated in the adolescent group. Corticosteroid was commonly used especially in generalized MG patients, and steroid-sparing immunosuppression use was limited. These findings support close monitoring of adolescent patients and highlight opportunities to optimize steroid-sparing management in juvenile generalized MG.

## Data Availability

The raw data supporting the conclusions of this article will be made available by the authors, without undue reservation.
