# Supplementary material for: Treatment patterns, intensive treatment, and hospitalization-related burden of juvenile generalized myasthenia gravis in Japan: a retrospective cohort study using a hospital-based claims database
Source: Front Neurol. 2026 Sep 15;17:1877931. doi: 10.3389/fneur.2026.1877931 (PMC13621577; doi:10.3389/fneur.2026.1877931)
Supplement: Supplementary file 1 [file Data_Sheet_1.pdf]

Supplementary Material

・Supplementary Table 1. Treatment trajectory over the whole follow-up period among patients with ≥1 year of follow-up.

1st treatment		2nd treatment		3rd treatment	
Treatment category	N (%)*	Treatment category	N (%)*	Treatment category	N (%)*
Number of patients who were treated 1st treatment	216 (100.0)	Number of patients who were treated 2nd treatment	124 (100.0)	Number of patients who were treated 3rd treatment	94 (100.0)
Cholinesterase inhibitors	116 (53.7)	None (End at the previous treatment)	44	-	-
		Oral steroid (glucocorticoid)	25 (20.2)	None (End at the previous treatment)	14
				Tacrolimus	2 (2.1)
				Cholinesterase inhibitors	1 (1.1)
				Oral steroid (glucocorticoid) + Tacrolimus	7 (7.4)
				Cholinesterase inhibitors + Oral steroid (glucocorticoid) + Tacrolimus	1 (1.1)
		Tacrolimus	2 (1.6)	None (End at the previous treatment)	2
		Cholinesterase inhibitors + Oral steroid (glucocorticoid)	32 (25.8)	None (End at the previous treatment)	3
				Cholinesterase inhibitors	11 (11.7)
				Oral steroid (glucocorticoid)	9 (9.6)
				Tacrolimus	2 (2.1)
				Cholinesterase inhibitors + Oral steroid (glucocorticoid) + Tacrolimus	6 (6.4)
				Oral steroid (glucocorticoid) + Tacrolimus	1 (1.1)
		Oral steroid (glucocorticoid) + Tacrolimus	9 (7.3)	None (End at the previous treatment)	2
				Tacrolimus	6 (6.4)
				Oral steroid (glucocorticoid)	1 (1.1)

		Cholinesterase inhibitors + Oral steroid (glucocorticoid) + Tacrolimus	4 (3.2)	None (End at the previous treatment)	2
				Cholinesterase inhibitors + Oral steroid (glucocorticoid)	1 (1.1)
				Oral steroid (glucocorticoid) + Tacrolimus	1 (1.1)
Oral steroid (glucocorticoid)	48 (22.2)	None (End at the previous treatment)	32	-	-
		Tacrolimus	1 (0.8)	Oral steroid (glucocorticoid) + Tacrolimus	1 (1.1)
		Oral steroid (glucocorticoid) + Tacrolimus	10 (8.1)	Tacrolimus	7 (7.4)
				Oral steroid (glucocorticoid)	2 (2.1)
				Oral steroid (glucocorticoid) + Other immunosuppressants	1 (1.1)
		Cholinesterase inhibitors + Oral steroid (glucocorticoid)	3 (2.4)	Oral steroid (glucocorticoid)	1 (1.1)
				Cholinesterase inhibitors + Oral steroid (glucocorticoid) + Tacrolimus	1 (1.1)
				Cholinesterase inhibitors + Oral steroid (glucocorticoid) + Cyclosporine	1 (1.1)
		Cholinesterase inhibitors + Oral steroid (glucocorticoid) + Tacrolimus	1 (0.8)	Oral steroid (glucocorticoid) + Tacrolimus	1 (1.1)
		Oral steroid (glucocorticoid) + Tacrolimus + Other immunosuppressants	1 (0.8)	Cholinesterase inhibitors + Oral steroid (glucocorticoid) + Tacrolimus + Other immunosuppressants	1 (1.1)
Tacrolimus	15 (6.9)	None (End at the previous treatment)	12	-	-
		Oral steroid (glucocorticoid)	1 (0.8)	None (End at the previous treatment)	1
		Cholinesterase inhibitors + Tacrolimus	1 (0.8)	Tacrolimus	1 (1.1)
		Oral steroid (glucocorticoid) + Tacrolimus	1 (0.8)	None (End at the previous treatment)	1
Oral steroid (glucocorticoid) + Tacrolimus	16 (7.4)	None (End at the previous treatment)	2	-	-
		Tacrolimus	11 (8.9)	None (End at the previous treatment)	1

				Oral steroid (glucocorticoid)	1 (1.1)
				Oral steroid (glucocorticoid) + Tacrolimus	9 (9.6)
		Cholinesterase inhibitors + Oral steroid (glucocorticoid) + Tacrolimus	2 (1.6)	Oral steroid (glucocorticoid) + Tacrolimus	2 (2.1)
		Cholinesterase inhibitors + Tacrolimus	1 (0.8)	Cholinesterase inhibitors	1 (1.1)
Cholinesterase inhibitors + Oral steroid (glucocorticoid) + Tacrolimus	9 (4.2)	None (End at the previous treatment)	1	-	-
		Tacrolimus	1 (0.8)	Oral steroid (glucocorticoid) + Tacrolimus	1 (1.1)
		Cholinesterase inhibitors + Tacrolimus	5 (4.0)	Cholinesterase inhibitors + Oral steroid (glucocorticoid) + Tacrolimus	5 (5.3)
		Oral steroid (glucocorticoid) + Tacrolimus	2 (1.6)	Cholinesterase inhibitors + Oral steroid (glucocorticoid) + Tacrolimus	2 (2.1)
Cholinesterase inhibitors + Oral steroid (glucocorticoid)	8 (3.7)	Oral steroid (glucocorticoid)	4 (3.2)	None (End at the previous treatment)	3
				Oral steroid (glucocorticoid) + Tacrolimus	1 (1.1)
		Cholinesterase inhibitors	3 (2.4)	Cholinesterase inhibitors + Oral steroid (glucocorticoid)	3 (3.2)
		Cholinesterase inhibitors + Oral steroid (glucocorticoid) + Tacrolimus	1 (0.8)	Cholinesterase inhibitors + Tacrolimus	1 (1.1)
Cholinesterase inhibitors + Tacrolimus	2 (0.9)	Tacrolimus	1 (0.8)	None (End at the previous treatment)	1
		Cholinesterase inhibitors + Oral steroid (glucocorticoid) + Tacrolimus	1 (0.8)	Oral steroid (glucocorticoid) + Tacrolimus	1 (1.1)
Cholinesterase inhibitors + Oral steroid (glucocorticoid) + Cyclosporine	2 (0.9)	None (End at the previous treatment)	1	-	-
		Cholinesterase inhibitors + Cyclosporine	1 (0.8)	Cholinesterase inhibitors + Oral steroid (glucocorticoid) + Cyclosporine	1 (1.1)

Treatment trajectories over the whole follow-up period among juvenile generalized MG patients with ≥1 year of follow-up after the index date. The table summarizes first-, second-, and third-line treatment categories recorded during follow-up. "None (End at the previous treatment)" indicates no further treatment change or escalation after the preceding regimen.

*Proportions are calculated using the number of patients who received the relevant treatment step (first-, second-, or third-line) as the denominator.

Supplementary Table 2. Hospitalization summary for each treatment within one-year follow up period for generalized MG patients.

		All	Children (0–9 years)	Adolescent (10–17 years)
Number of target cases		261	125	136
Length of IVIg-associated hospitalizations per hospitalization (days)				
	n	48	13	35
	mean (SD)	29.7 (61.3)	22.6 (18.6)	32.4 (71.0)
	median	9.5	16.0	7.0
	q1 - q3	5.0 - 28.5	7.0 - 28.0	5.0 - 29.0
	min - max	2 - 407	4 - 61	2 - 407
Length of PE-associated hospitalization per hospitalization (days)				
	n	19	1	18
	mean (SD)	43.6 (92.2)	26.0 (-)	44.6 (94.8)
	median	19.0	26.0	12.0
	q1 - q3	2.0 - 32.0	26.0 - 26.0	2.0 - 32.0
	min - max	2 - 407	26 - 26	2 - 407
Length of Steroid pulse therapy-associated hospitalization per hospitalization (days)				
	n	29	4	25
	mean (SD)	35.3 (28.3)	46.0 (21.0)	33.6 (29.3)
	median	29.0	49.5	28.0
	q1 - q3	19.0 - 38.0	29.0 - 63.0	15.0 - 37.0
	min - max	4 - 123	20 - 65	4 - 123
Length of ICU/respirator-associated hospitalizations per hospitalization (days)				
	n	13	1	12
	mean (SD)	62.5 (110.2)	28.0 (-)	65.3 (114.6)
	median	15.0	28.0	14.0
	q1 - q3	10.0 - 53.0	28.0 - 28.0	9.5 - 78.0

	min - max	6 - 407	28 - 28	6 - 407
Hospitalizations with first intensive treatment recorded within 3 days after the hospitalization start date		56	13	43
	Time from first intensive treatment to the end of hospitalization:			
	<=7 days	27 (48.2)	4 (30.8)	23 (53.5)
	>7 to <=14 days	8 (14.3)	2 (15.4)	6 (14.0)
	>14 to <=28 days	15 (26.8)	6 (46.2)	9 (20.9)
	>28 days	6 (10.7)	1 (7.7)	5 (11.6)
	Time from last intensive treatment to the end of hospitalization:			
	<=7 days	39 (69.6)	6 (46.2)	33 (76.7)
	>7 to <=14 days	8 (14.3)	2 (15.4)	6 (14.0)
	>14 to <=28 days	7 (12.5)	4 (30.8)	3 (7.0)
	>28 days	2 (3.6)	1 (7.7)	1 (2.3)
Length of first hospitalization per patient (days)				
	n	135	73	62
	mean (SD)	20.4 (41.6)	14.3 (24.0)	27.5 (55.0)
	median	8.0	6.0	13.0
	q1 - q3	3.0 - 23.0	2.0 - 17.0	5.0 - 29.0
	min - max	1 - 407	1 - 169	1 - 407
Length of first hospitalization per patient (length category)				
	<1 week	59 (43.7)	38 (52.1)	21 (33.9)
	1-2 week	26 (19.3)	14 (19.2)	12 (19.4)
	2-4 week	20 (14.8)	9 (12.3)	11 (17.7)
	>4 week	30 (22.2)	12 (16.4)	18 (29.0)
Minimum length of hospitalization per patient (days)				
	n	135	73	62
	mean (SD)	16.9 (40.9)	10.8 (22.6)	24.1 (54.5)
	median	5.0	4.0	9.0
	q1 - q3	2.0 - 16.0	2.0 - 8.0	4.0 - 22.0
	min - max	1 - 407	1 - 169	1 - 407
Minimum length of hospitalization per patient (length category)				
	<1 week	73 (54.1)	49 (67.1)	24 (38.7)

	1-2 week	23 (17.0)	9 (12.3)	14 (22.6)
	2-4 week	20 (14.8)	8 (11.0)	12 (19.4)
	>4 week	19 (14.1)	7 (9.6)	12 (19.4)
Maximum length of hospitalization per patient (days)				
	n	135	73	62
	mean (SD)	24.6 (42.3)	18.6 (24.6)	31.6 (55.9)
	median	13.0	10.0	14.5
	q1 - q3	6.0 - 29.0	4.0 - 22.0	7.0 - 33.0
	min - max	1 - 407	1 - 169	2 - 407
Maximum length of hospitalization per patient (length category)				
	<1 week	40 (29.6)	26 (35.6)	14 (22.6)
	1-2 week	32 (23.7)	16 (21.9)	16 (25.8)
	2-4 week	27 (20.0)	16 (21.9)	11 (17.7)
	>4 week	36 (26.7)	15 (20.5)	21 (33.9)

SD: Standard Deviation

Percentages for length of hospitalization categories are calculated using the number of patients with hospitalization as the denominator.

Supplementary Figure 1. Sankey diagram showing treatment trajectory over the entire follow-up period among patients with ≥1 year of follow-up for children aged 0-9 years (A) and adolescents aged 10-17 years (B).

(A) Children aged 0-9 years

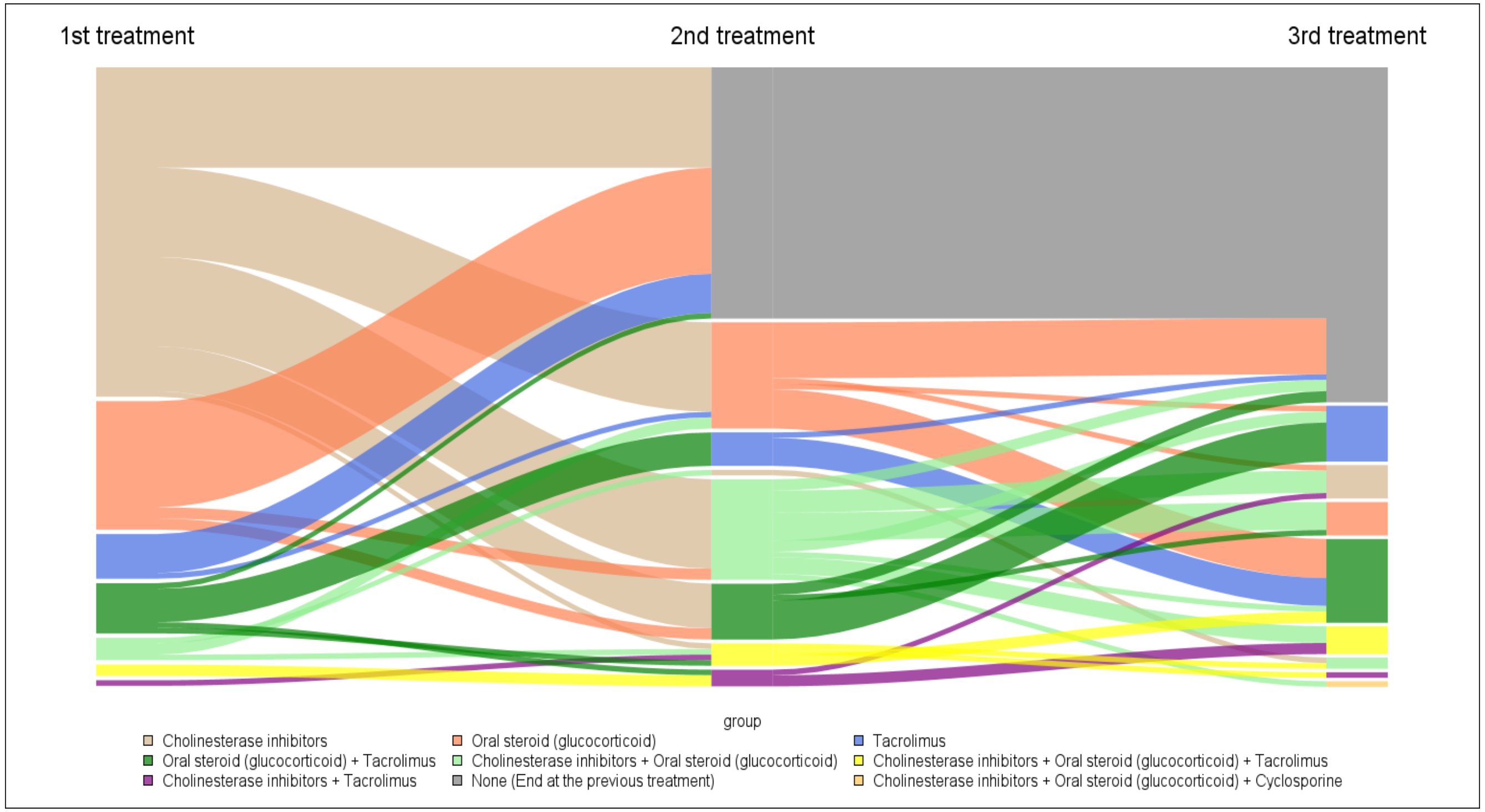

(B) Adolescents aged 10-17 years

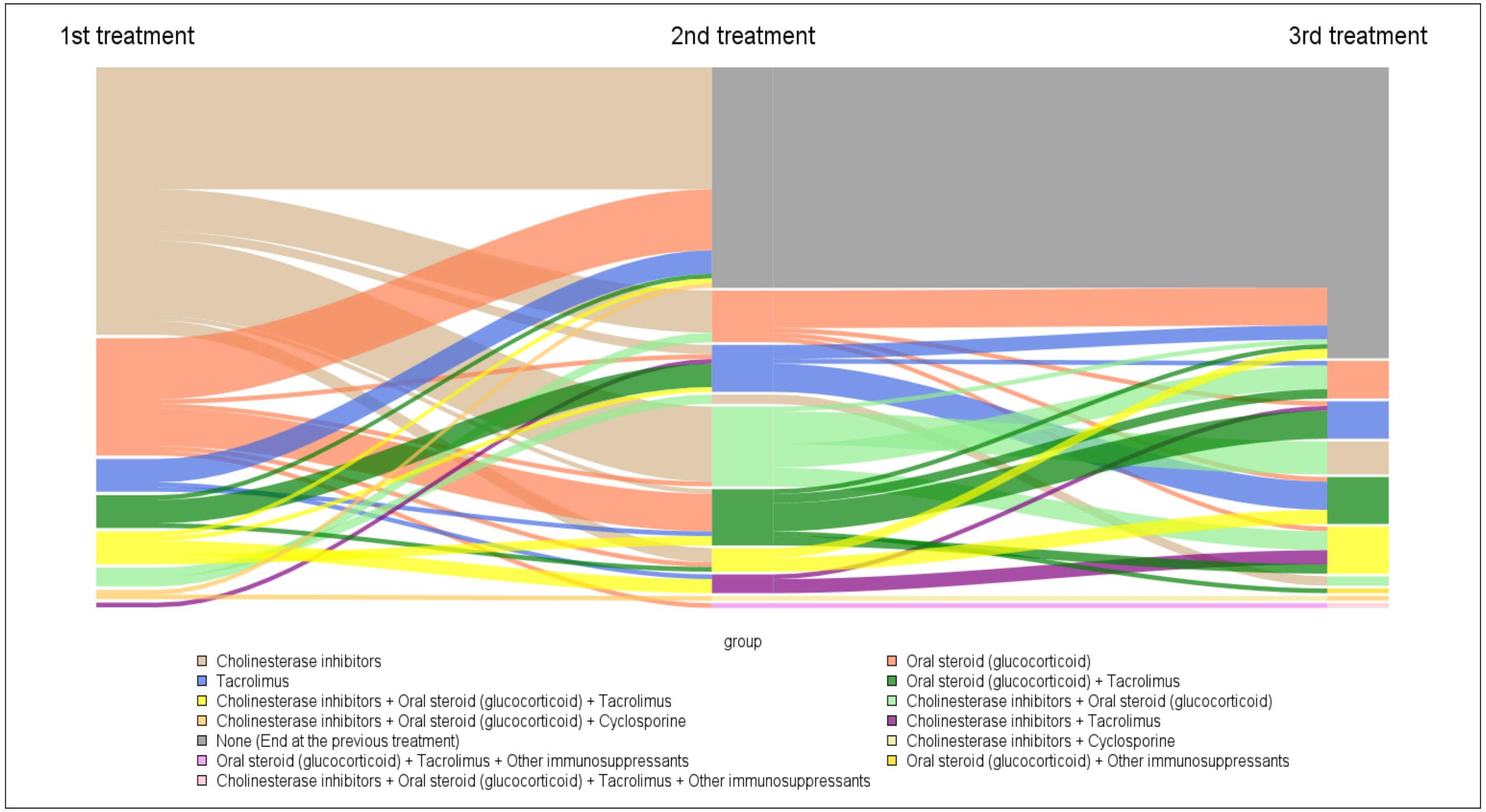

Different colors represent distinct treatment regimens, including single and combination therapies, as detailed in the legend. The three horizontal columns (1st, 2nd, and 3rd treatments) illustrate the chronological sequence of treatment modifications rather than specific intervals. The gray area indicates the discontinuation of treatments.
